# The Pedagogical Model of Hybrid Teaching: Difficulties of University Students in the Context of COVID-19

**DOI:** 10.3390/ejihpe11040096

**Published:** 2021-10-22

**Authors:** Alejandro Lorenzo-Lledó, Asunción Lledó, Alba Gilabert-Cerdá, Gonzalo Lorenzo

**Affiliations:** Department of Development Psychology and Teaching, Faculty of Education, University of Alicante, 03690 San Vicente del Raspeig, Spain; asuncion.lledo@ua.es (A.L.); alba.gilabert@ua.es (A.G.-C.); glledo@ua.es (G.L.)

**Keywords:** COVID-19, hybrid teaching, dual teaching, online learning, university education, difficulties, innovation

## Abstract

The global pandemic caused by COVID-19 has led to changes in many areas, with educational scenarios being affected. In this sense, university education has undergone significant changes owing to the impossibility of following the fully face-to-face mode of teaching. Given this situation, the general objective of this study is to analyze the university educational scenario in the context of COVID-19 and, more specifically, to identify the difficulties perceived by students. Using a mixed quantitative–qualitative methodological approach, an ad hoc questionnaire was designed, and data were collected from a sample of 238 students of the Bachelor’s Degree in Teaching during the 2020/2021 academic year. The results obtained have shown that students have experienced numerous difficulties in adapting to the hybrid teaching model. In this sense, it is worth highlighting the decrease in motivation, the feeling of loneliness, technical connection problems, and less interaction with the teaching staff and other students. The degree of satisfaction with the teaching received is also moderate. As a conclusion, it can be stated that the difficulties identified recommend the introduction of actions to improve the application of the teaching model implemented in favor of university excellence.

## 1. Introduction

In the second decade of the 21st century, a pandemic caused by the COVID-19 virus has taken the entire planet by surprise, and no one could have imagined the real impact and repercussions on all levels [1,2]. Its effects have shaken socio-economic structures at the global level, because, since the confirmation of its existence in January and February 2020, the measures to stop its spread involved great efforts and an unimaginable cost to health systems [3] with global and fulminant effects, in what the World Health Organization (WHO) has called the biggest crisis since the Second World War [4].

In this sense, in the field of education, there was a change from the conventional model of face-to-face classes to a completely virtual model [5]. In Spain, Royal Decree 463/2020 of 14 March [6] led to the suspension of face-to-face teaching at all levels of education, including universities. The measures taken were in line with the urgency and not with a priori planning to teach a subject with a fully online methodology [7]. Higher education institutions worldwide have had to adapt to the virtual modality and, with it, all their academic, administrative, and civil servants, so that the learning experience is as similar as possible to the face-to-face experience of students [8].

Proof of this is that, at the beginning of the pandemic, Spanish universities considered various formulas to respond to the problems that suddenly arose in teaching, but the situation has continued, and non-face-to-face or hybrid modalities have been consolidated, which have required the adaptation of the different educational agents. Nevertheless, virtual education in the university environment can be considered acceptable because of the fact that universities already had virtual teaching and learning platforms incorporated into their education systems [9].

The decisive change was due to the impossibility of continuing with face-to-face classes, as the restrictive measures to prevent the spread of the virus made it impossible to teach classes synchronously with all the students physically present in a classroom. Similarly, it has been suggested that the 21st century student must acquire the skills and knowledge that will enable them to face the future challenges of organisations [10], and that is why they must face this new education system. Despite this, for UNESCO [11], there is a digital divide that forms part of the obstacles to the development of a knowledge society, as it must be a shared good, which is limited by different barriers such as the social, economic, or family dimension.

In this line, hybrid teaching and blended learning, also known as dual learning, arise, presented as a solution to the requirements of today’s society, being defined [12] as one that combines face-to-face instruction and online instruction mediated by information and communication technologies. Along the same lines, the term blended learning has also been coined [13] as a modality in which teaching and learning take place both in physical spaces and in virtual or online environments. This pedagogical model provides the possibility of continuity in the teaching–learning process as it can be seen as the expansion and spatio-temporal continuity (face-to-face and non-face-to-face, synchronous and asynchronous) in the learning environment [14].

It should be noted that this hybrid system has been applied in some cases as a flipped classroom approach [15], with this situation being seen as an emergency remote teaching educational scenario [16], far from being distance or online teaching per se. This suggests that incipient models of what will be known today as blended, hybrid, or blended learning were already being developed in some university educational experiences, and that this pandemic situation has highlighted their debate in the clarification of these assumptions.

The University of Alicante in Spain, following and respecting the health instructions at national and regional level to preserve the safety conditions, opted in the academic year 2020/2021 for dual teaching, also guaranteeing optionally for the university students of face-to-face teaching, whenever it can be carried out. This dual teaching modality is characterized as hybrid teaching, where students have the option of attending the assigned classes in person and, for the rest of the classes, they can follow the programmed content online. This pedagogical model, supported by technology, was implemented through the UACloud virtual platform, in which there was a virtual classroom accessed by both teaching staff and students to carry out the teaching and learning process.

This reality of new online educational scenarios has affected all aspects of the teaching–learning process, accelerating the process of digital transformation. In addition to the possible problems caused by the digital divide, there are also the training requirements for both teachers and students in the face of non-face-to-face educational scenarios. This is the reason for the substantial change that teaching–learning has had to undergo, from face-to-face to virtual, requiring teaching to be adapted, starting with the new planning of subjects and exams to adapt to university spaces [17]. In this vein, the online learning context offers a distinctive pedagogical approach as opposed to face-to-face learning that involves an adjustment and willingness to engage in an effective learning experience [18].

As a result of the changes brought about by the global pandemic, an important scientific production on the consequences of COVID-19 in the educational context has been emerging at the research level. Thus, the aim has been to address, from different perspectives, the problems that have arisen with the adaptation to the new teaching and learning scenario. Thus, the authors of [19,20,21] have addressed the new educational scenarios created and the difficulties and weaknesses encountered, delving deeper into the students’ vision. From this, they have sought to extract indicators for the change of teaching strategies and practices, which promote more effective teaching and meet the needs of the student body. In the same line, the authors of [22] discussed the opinion of students on different aspects of online education, expressing that this modality is useful during the pandemic to continue with the study, despite detecting numerous obstacles. Some authors were struck by the loneliness experienced by students in online education, reflecting that they miss the help they receive from their peers in classrooms and laboratories and access to the library [23]. Likewise, the authors of [24] investigated the technological resources of the student body to cope with the change in education, and it was found that the emergence of COVID-19 has made the existing digital divide between urban and rural areas more visible. Also, the authors of [25] compare university students’ learning and their experiences with the teaching–learning environment in general and during online study because of the COVID-19 pandemic. Thus, it was found that students have had difficulty managing their time and more fragmented knowledge has been generated. It is added that students who tend to relate ideas, but with less ability to manage time and effort, have manifested a certain exhaustion with online teaching.

Another problem dealt with was the effects of the recent new teaching method on teachers, showing that it has led to an improvement in their digital skills and professional vision of this method of teaching classes [22]. Furthermore, research has been conducted on the use of technological tools by university students, and it has been found that it has led to the development of digital skills to improve their professional training, finding in technological tools an exceptional resource for their personal growth [26]. Online teaching–learning activities during the pandemic period have also been analyzed, leaving evidence that, even though, in the university, there was already a significant infrastructure in place to migrate from face-to-face to online teaching, there was no preparation for the change [27]. Other studies [28,29] have reflected virtues in the online teaching model, by making possible the creation of real situations that could be solved by the student as if they were in the face-to-face classes. Nevertheless, the authors of [30,31] have addressed the weaknesses of the adopted teaching modality, concluding that the learning environments created have had a negative impact on the students’ scheduling and management of the study and on interactions with teachers and other students. For their part, the authors of [32] delve comparatively deeper into the teaching strategies applied and student satisfaction in two countries, detecting that the teacher–student relationship is negatively affected by the impossibility of maintaining direct and close contact in the classroom.

Research has also been conducted on the impact of the pandemic on the mental domain [33,34] and, more specifically, the stress, depression, and anxiety processes triggered in students in the context of COVID-19 [35,36,37,38]. Suicide attempts [39] and the degree of resilience among students, which is higher among university students living alone, have also been tackled [40]. Moreover, the difficulties of lack of motivation and concentration and the emergence of negative emotions, which has been a weakness in online teaching, have also been treated in this direction [23]. In agreement, the concern on the part of the student body of the negative effects on their future professional careers has been studied [41]. The use of the cell phones for learning during COVID-19 was also analyzed from the students’ perspective and was considered very useful to recover studies during the pandemic and to improve the teaching process [42].

Based on the above background, it was considered necessary to investigate the new context of university teaching. In this sense, the present work arises from an Innovation and Research Project in university teaching granted by the Vice-Rectorate for Quality and Educational Innovation through the Education Sciences Institute (ESI) of the University of Alicante (Networks-I3CE Program for Research in University Teaching 2020–2021). The general objective is to analyze the university scenario in the context of COVID-19. This general objective underlies the following specific objectives:

1. To find out the students’ preference regarding the mode of university education.
2. To identify the difficulties encountered in the teaching received.
3. To examine the experience of the difficulties encountered in the teaching received.
4. To detect the perception of the impact on academic performance and the degree of satisfaction with the teaching received in times of COVID-19.

## 2. Materials and Methods

The study is presented with a mixed quantitative and qualitative approach [43], with a non-experimental and descriptive design. On the other hand, the data were collected at a specific point in time, making it a cross-sectional study [44].

### 2.1. Research Context and Participants

This study is part of an innovation and research project in university teaching granted by the Vice-Rectorate for Quality and Educational Innovation and the Education Sciences Institute (ESI) of the University of Alicante for the academic year 2020/2021. These projects are awarded to develop research to further innovation and improvement in higher education.

The context in which the research was carried out was the Faculty of Education at the University of Alicante, and participants were selected using a non-probabilistic casual sampling technique, which is characterized using as a sample individuals who are easily accessible [45]. The sample consisted of 238 students, 65.3% of whom were from the Degree in Primary Education and 34.7% from the Degree in Early Childhood Education. In turn, 82% were women and 18% were men. In terms of year of study, 25.6% were first-year students, 38.9% were second-year students, 23.4% were third-year students, and 12.1% were fourth-year students. The age of the participants ranged from 17 to 53 years (M = 20.71; SD = 4.17). In addition, 52% took the subjects online, 44.7% in the dual mode, and 3.3% in the fully face-to-face mode.

### 2.2. Instrument

For the collection of information, an ad hoc questionnaire was designed, taking as a reference the bibliographical review carried out in the first phase of the study. In a previous phase, a first version of the questionnaire containing 33 items was drawn up. In order to obtain adequate content validity, the expert judgement technique was used, considered as the most appropriate mechanism in the evaluation of ICT [46,47]. Twelve experts participated in this process, among whom there were eight lecturers and four full professors, who had coordinated innovation projects in higher education at the university and outside the university. For the quantitative assessment of the items, the Aiken V coefficient [48], which has the formula V = (X − 1)/k, was chosen. This coefficient is one of the most widely used techniques to quantify the content validity or relevance of the item with respect to a content domain in N judges, whose magnitude ranges from 0.00 to 1.00; the value 1.00 is the highest possible magnitude, indicating perfect agreement among the judges with respect to the highest content validity score [49].

All experts responded to the evaluation of the questionnaire on the basis of the template provided. Items are considered valid with a significance level of greater than or equal to 0.80 [50]. However, three of the items obtained values of 0.54, 0.72, and 0.46 and were thus eliminated. The rest of the items obtained values between 0.80 and 1. Therefore, the final version consisted of 30 items. This means that a good content validity can be affirmed.

The questionnaire is structured in several parts, as follows:

− First part: participants’ identification data.
− Second part: student preferences with regard to the mode of university education and resources available (closed-ended questions).
− Third part: difficulties encountered by university students in the teaching received (closed-ended questions).
− Fourth part: experience of difficulties encountered in the teaching received (open-ended questions).
− Fifth part: general evaluation of the teaching received (closed and open-ended questions).

Prior to the dissemination of the questionnaire, a pilot test was carried out with 38 students in order to verify the correct understanding of the questions by the students. After collecting the comments made, the final version of the instrument was obtained.

Regarding reliability, the Cronbach’s alpha coefficient of the Likert scale designed for the difficulties encountered was calculated, obtaining a value of 0.820, which is a good reliability value [51].

### 2.3. Procedure

The data collection procedure was carried out during the 2020/2021 academic year. The research teaching team was responsible for disseminating the questionnaire among the students. The questionnaire was completed using Google Forms and the objectives of the research and the voluntary nature of participation were previously informed. In this sense, it was informed that participation was anonymous, and consent was obtained from the students. Any personal data that did not respect confidentiality were excluded and the information collected was used exclusively for the purposes of this research.

### 2.4. Data Analysis

Once the data collection procedure was planned and implemented, the data obtained were analyzed. For the quantitative analysis, the SPSS 22 statistical program (IBM Corp., Armonk, NY, USA) was used, including descriptive statistics with frequencies, percentages, and means. For the open-ended questions, a qualitative analysis of the content was carried out, ordering the responses based on the relevant categories identified.

## 3. Results

The results of the study are presented below, structured in relation to the analyzed variables and the proposed objectives.

### 3.1. Student Preference for the Mode of University Education

**Figure 1:**
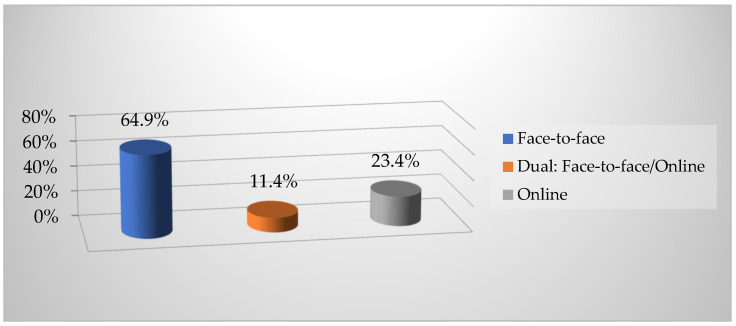
Percentage of preference in university education.

Figure 1 below shows the results on the students’ preferred mode of university education.

As can be seen, 64.9% of students prefer face-to-face teaching, compared with 23.7% who prefer dual or hybrid teaching.

### 3.2. Difficulties Encountered in University Teaching during COVID-19

In relation to the perceived difficulty of teaching during COVID-19, 72.8% indicated that they found it difficult and 27.2% that they did not.

Table 1 below shows the results obtained in relation to the difficulties encountered by university students in the teaching they received.

The results show an orientation of the percentages towards the response categories identified as considerable difficulty and total difficulty in more than half of the items. The highest degrees of perceived difficulty are indicated in items 9 and 10, followed by items 2, 5, 7, and 8. In addition, means above four are observed for most of the perceived difficulties.

### 3.3. Experience of Difficulties Encountered in the Teaching Received

The results of the open-ended questions on adaptation to personal situations and adaptation to teaching and learning processes are presented below.

#### 3.3.1. Experience of Difficulties Encountered in Adapting to Personal Situations

The participating students have experienced fewer difficulties in adapting the dual learning modality to their personal situations. In this sense, it is indicated that, “This type of dual education has made it possible to reconcile studies with work” and “This type of dual education has favored family reconciliation”. On the other hand, it is highlighted that, “It is a flexible modality that adapts to personal situations, and we have to take advantage of it” and “It has allowed us to work more autonomously”. With regard to time management and the economic cost of learning, it is stated that, “during this course less time has been lost as we have not had to travel” and “This dual modality is more economical for the university student”.

#### 3.3.2. Experience of Difficulties Encountered in Relation to the Teaching and Learning Process

The results show that students have experienced difficulties related both to the didactic and methodological aspects of teaching and to the technological elements of dual teaching. In this sense, it is indicated that, “It has worked better than expected although there have been quite a few technical problems”, and “It is easy to adapt to the dual classroom although some technical problems were present”. In addition, different attitudes of the teaching staff were noted: “There are teachers who have made great efforts to motivate, but learning is quite passive”. On the other hand, aspects of the performance and the new role of the teaching staff were identified as having a direct impact on learning: “The teaching staff have tried to guide us but, in some aspects, it has been complex as they have not been present in person”, and “The teaching staff have been more concerned with attending to those who are at home than in class”. With regard to interaction and communication, students experienced difficulties in following the classes due to the work of the teaching staff: “It is very important that the teaching staff communicate well to motivate us, and on many occasions, this has not happened”. The teachers’ lack of understanding of the difficulties experienced by the pupils is also expressed: “The lack of empathy when we don’t wear the camera is a great difficulty and, more significantly, it will also be a difficulty for the teachers”.

The results obtained also reflect shortcomings in adapting to the new educational scenario. In this sense, it is stated that, “The syllabuses and materials have not been adapted to this type of dual education, and this has made it very difficult for us to follow the classes”. Likewise, the results show difficulties in motivating students and making them feel involved in their learning. In this regard, it is stated that, “The classes have become very passive and with few practices to interact” and it is added that “This teaching requires a lot of attention and concentration, as we are not in a suitable study environment” and “There are many distractions in this type of teaching that can harm our grades”. Another negative experience is the lack of coordination between face-to-face and online classes, indicating that, “I have an online class at one hour and then I have another face-to-face class and I don’t have time to move” and “I can’t follow the face-to-face and online classes that I am given in a continuous way”.

### 3.4. Perceived Impact of Dual Education on Academic Performance and Level of Satisfaction

**Figure 2:**
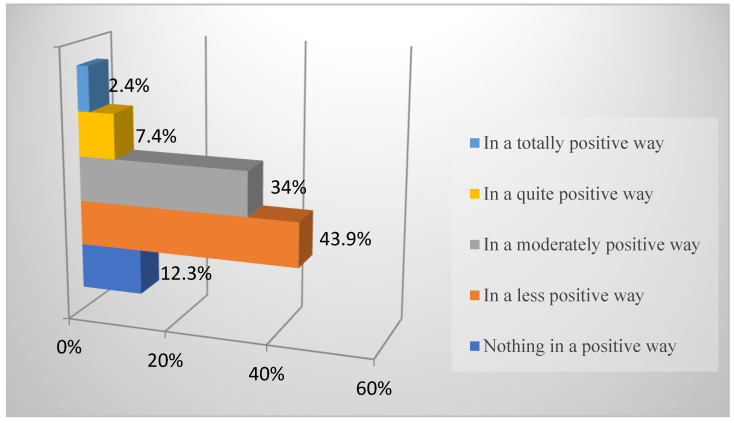
Percentages of the impact of non-face-to-face teaching on academic achievement.

In relation to the results obtained on the students’ perception of the impact that the teaching received may have on academic performance, Figure 2 is presented.

The results indicate that 56.2% of the students consider the impact of the hybrid mode of education on their academic performance to be not positive or less positive. In contrast, 34% consider the impact to be moderately positive.

**Figure 3:**
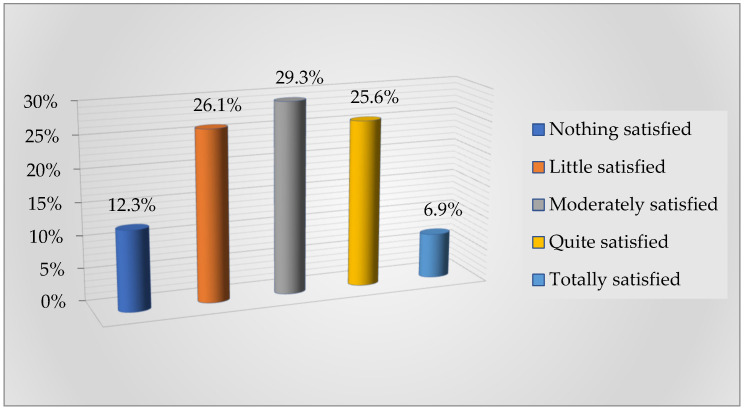
Degree of satisfaction of university students with the teaching.

The results obtained in regard to the degree of satisfaction of university students with the teaching received are shown in Figure 3.

As can be seen, the findings show that 55.4% of students are little or moderately satisfied, while 6.9% say they are totally satisfied with the teaching received.

## 4. Discussion

The study presented set out to analyze the university educational scenario in the context of COVID-19, in which a hybrid teaching model has been implemented. In this line, special attention has been paid to the identification and assessment of the difficulties derived from adapting to teaching with online classes and the degree of satisfaction of the students with the teaching received. Firstly, in relation to preferences regarding the teaching modality, most students prefer face-to-face classes. This finding is in line with other studies [19,22], which found that 65% of students prefer face-to-face teaching. The fact that students value face-to-face classes, or hybrid teaching, more positively than fully online classes may be because it is considered a more effective modality for the resolution of doubts, the development of learning, and participation and interaction [10].

If we look at the difficulty experienced by the students, 72.8% described the adaptation to the new teaching modality as difficult, identifying various difficulties. In this sense, it is worth highlighting, in the first place, the difficulties in interacting with the teaching staff and classmates, in accordance with the results obtained by [19,21], who found percentages of students above 80% who expressed having experienced a feeling of lack of communication and frustration and reject the effectiveness of the adaptation carried out by the university. In addition, technological difficulties have been detected in accessing the virtual classroom. This finding is corroborated by [19], who found that students, on a scale of one to ten, were highly affected by connectivity in classes and had problems connecting to the Internet to hand in assignments. The findings show that the monitoring of learning in hybrid teaching has led to disadvantages for students, which, according to [20,52], are influenced by a lack of training and technological resources and a mentality that is reluctant to change and innovation. On the other hand, insufficient guidance and guidance from teaching staff has been found. In this sense, another study [53] has shown the lack of guidance from the universities and the lack of flexibility of the teaching staff, pointing out that communication with the teaching staff and their role as guides and facilitators of learning is essential for the success of the distance learning model. On the other side, it is worth mentioning the difficulty perceived by students to find motivation in learning and not to feel a sense of loneliness. In accordance with this finding, the authors of [54] found anxiety and mental health disorders in students during the pandemic, correlated with delays in academic activities, which were only lessened by social support. In the same vein, the authors of [22] found increased stress among students. Furthermore, a higher degree of flexibility and autonomy also leads to a higher risk of dropping out of school owing to feelings of loneliness and responsibility in time management, a challenge to overcome associated with the online context [55].

Concerning the experience of the students, it should be noted that, despite the difficulties encountered, it was perceived that the teaching received has enabled them to adapt better to the rhythms of work and to make their working life compatible with their studies. In this regard, in view of the situation caused by COVID-19, it has been positively identified that, in this aspect, a learning environment has been implemented that allows for greater learning achievement, with the degree to which the students’ basic psychological needs are satisfied being a determining factor [56]. Similarly, the authors of [57] found that 90% of students combine online classes with family and professional tasks, which is seen as an opportunity to advance in the implementation of a new academic paradigm based on e-learning. However, it should be borne in mind, as noted above, that this fact is accompanied by the decrease in motivation experienced by students, which undoubtedly requires the adoption of proactive action strategies that reorder the educational teaching process [58].

Another difficulty experienced by students is the decrease in interaction during online learning. Along these lines, the authors of [59,60] point out that interaction between the student and the teacher is a fundamental factor in breaking the feeling of isolation by generating a sense of belonging, as well as improving academic performance. In keeping with the promotion of autonomy that has been found in this mode of teaching, several studies [61,62] have considered online teaching to be particularly favorable for learning at university, as it facilitates different and flexible learning scenarios. To fulfil this purpose, it is necessary to change the role of the lecturer from a mere transmitter of knowledge to a guide, tutor-mentor [61], with the student becoming the main one responsible for his or her learning [63,64]. It has been found that this change has not yet fully taken place. Thus, the study has shown that there are several aspects that need to be improved, such as poor advice and guidance from the teacher, poor time management on the part of the student, and the tendency to develop a more passive learning style. Along these lines, it was found that the teaching materials were not adapted, as also revealed by [65], who found that students perceived that the teachers did not use resources geared towards virtual teaching, but rather that previous tools were reproduced.

Regarding the students’ assessment of the teaching received, there is concern about the negative repercussions that this new educational scenario may have on academic performance. Thus, only 9.8% of students consider that the distance learning mode will have a fairly or totally positive impact on their performance. Analogous results were obtained by [65], who detected percentages of students above 55% who state that learning in this modality is lower and that online classes cannot replace face-to-face classes. They also [27] displayed students’ perception of the decline in the quality of teaching and its negative influence on their performance. On the other part, it is worth noting that students are not very satisfied with the teaching received, with the highest percentage of students being moderately or not very satisfied. Following this line, the authors of [19] found that 53.3% of the students were not very satisfied with the virtual teaching received and the situation experienced is considered to have a negative influence on future employment. The result obtained should be considered, because, as stated by [66], student satisfaction is a valuable indicator in the evaluation of an educational center and contributes to the better management of universities, as it is an increasingly relevant factor in teaching processes, skills, and attitudes.

## 5. Conclusions

The study presented has shown that the change to hybrid teaching in the context of COVID-19 has entailed difficulties for the students. In this sense, based on the objectives set out, the following conclusions are indicated:

1. After the experience, the students prefer the previous modality of face-to-face classes.
2. The process of adaptation and monitoring of hybrid teaching has been perceived as difficult by the students. In this line, difficulties have been found both in the teaching-learning process and in the technological field.
3. For the students, hybrid teaching has favored a more autonomous and flexible learning process, but difficulties have also been experienced in the interaction with the teaching staff and classmates and during the learning of the content.
4. The majority of students consider that hybrid teaching will not have a positive impact on their academic performance and their degree of satisfaction with the teaching received is moderate.

When evaluating the findings obtained, some limitations of the study should be taken into account. In this regard, the participating sample is not representative, and the results cannot be extrapolated to other faculties. On the other hand, the pandemic, being an unforeseen situation for which an urgent response was given, may have involved extraneous variables that could have influenced the results extracted from the sample.

As future lines of research, it is considered necessary to follow up on the evolution of the teaching implemented as we progressively return to face-to-face classes. On the other hand, it is worth investigating the repercussions that this pedagogical model will have on the training and future professional performance of university students. It is also of interest to investigate the changes that will be made to respond to emerging developments, and the possible consolidation of the measures implemented that may lead to a stable hybrid teaching model. Likewise, it is deemed advisable to complement the results presented with other results drawn from other objectives and analysis techniques, such as correlational analyses, which complement the findings of this study.

This study is a first diagnosis of the situation experienced in university teaching in times of COVID-19 and should lead to reflection on the strategies used in different aspects of dual or hybrid teaching in order to finalize proposals for improvement in the short and medium term. What emerges from this study is the opportunity presented to implement, even if it arises from an unforeseen situation, new educational scenarios that incorporate new methodologies that do not focus so much on face-to-face teaching and more on the promotion of true autonomous work, as prescribed by the current European Higher Education Area.

On a practical level, the conclusions obtained recommend a series of decisions to be taken as a protocol for action in different areas, depending on the weaknesses detected in the hybrid teaching model:

− Teachers. Design of training plans aimed at teachers, which contribute to improving their communicative, didactic, and digital competences. In this way, the intention is that teachers do not limit themselves to reproducing the face-to-face pedagogical model in hybrid teaching and carry out didactic innovations in line with the new educational scenario.
− Universities. Coordination to avoid the overlapping of face-to-face and online classes, application of adequate attention and advice for students, and economic provision to guarantee the necessary material resources and optimize the pedagogical model implemented.
− Methodology. Implementation of strategies for more active, motivating, and autonomous learning with the continuous guidance of the teacher. Likewise, asynchronous spaces should be enabled for collaborative learning and the resolution of doubts through forums, in which both students and teachers can participate, and which minimize the possible loneliness of the student in their learning.
− Development of content. Planning of the activities to be carried out synchronously in the virtual spaces during the theoretical content classes. Subsequently, in the virtual sessions, the teacher must address the key issues of each subject and, finally, the student must develop the content autonomously. On the other hand, in relation to the practical sessions, cooperative groups and face-to-face sessions should be encouraged, although synchronous online sessions should be included for those practical contents which, owing to their nature, allow for greater flexibility.

With the recommendations indicated above, the difficulties experienced by students could be reduced, but also they could take advantage of the situation to incorporate academic actions in a stable way that complement face-to-face teaching and give rise to a more innovative, flexible, and quality pedagogical model for university teaching.

## Figures and Tables

**Table 1:** Difficulties perceived by university students in the dual teaching received.

Items	ND		LD		MOD		QD		TD		M
	*f*	*%*	*f*	*%*	*f*	*%*	*f*	*%*	*f*	*%*	
1. The personal technological means used.	71	30.0	110	46.1	21	9.0	14	7.8	21	9.0	2.18
2. The connection to the virtual classroom.	5	2.1	10	4.2	21	8.9	59	24.9	143	59.9	4.36
3. Coordination with face-to-face and non-face-to-face/online classes.	21	8.7	24	10.0	48	20.2	53	22.4	92	38.7	3.72
4. Interaction with classmates.	6	2.5	15	6.1	24	10.1	79	33.1	115	48.2	4.18
5. Interaction with teachers.	4	1.8	5	2.3	17	7.0	84	35.2	128	53.7	4.37
6. The connection with other platforms such as meet, zoom, and so on.	33	13.7	30	12.8	69	28.9	48	20.3	58	24.3	3.29
7. Time management for study and daily work.	3	1.3	4	1.5	7	3.1	115	48.3	109	45.8	4.36
8. Individual guidance and counselling by teachers.	2	0.9	3	1.4	31	13.1	96	40.3	105	44.3	4.26
9. The feeling of loneliness in the face of the learning to be done.	2	1.0	3	1.1	24	10.0	48	20.1	161	67.8	4.53
10. Motivation in the face of the learning that was taking place.	2	0.8	3	1.1	13	5.5	48	20.1	173	72.5	4.62

Note: ND = no difficulty; LD = little difficulty; MOD = moderate difficulty; QD = quite difficulty; TD = total difficulty; M = mean.

## Data Availability

The data presented in this study are available upon reasonable request to the corresponding author.

## References

[1] Grande-de-Prado M.G., Peñalvo F.J.G., Corell A., Abella-García V. (2021). Evaluación en Educación Superior durante la pandemia de la COVID-19. Campus Virtuales.

[2] Ramos C. (2020). COVID-19: La nueva enfermedad causada por un coronavirus. Salud Pública de México.

[3] Nando M.A., Nando M.A., Muñoz V.M.R., Ramos M.L.R. (2020). Una nueva forma de educar en educación superior. La pandemia De La COVID-19 Como Oportunidad Para Repensar la Educación Superior en MÉXICO.

[4] Zaar M.H., Ávila M.B.G. (2020). El COVID-19 en España y sus primeras consecuencias. Revista Brasileira de Geografia Econômica.

[5] Lebrón J.A., Jiménez-Rosado M., Ostos F.J., Perez-Puyana V. (2021). Comparativa de la enseñanza presencial y no presencial de asignaturas científicotécnicas en la Universidad de Sevilla. Afinidad.

[6] Royal Decree 463/2020, of 14 March, Declaring a State of Alarm for the Management of the Health Crisis Situation Caused by COVID-19. BOE, 67, of 14 March 2020. https://www.boe.es/eli/es/rd/2020/03/14/463/con.

[7] García-Peñalvo F.J., Corell A., Abella-García V., Grande M. (2020). La evaluación online en la educación superior en tiempos de la COVID-19. EKS.

[8] Ferro E.F., Gutiérrez N., Añasco N., González M., Villafaña L., Flores P.G., Cid F.M. (2021). Satisfacción de las clases online de estudiantes de educación física de una universidad de chile en tiempos de pandemia. EmásF Revista Digital de Educación Física.

[9] Villalta D.A.T., Zavala J.O.A., Pérez A.F.F., Garzón M.E.U. (2021). Impacto de la enseñanza virtual en el rendimiento académico de estudiantes de estadística con diferentes estilos VAK de aprendizaje. Rev. Conrado.

[10] Sousa S., González M.J.P., Sepúlveda J.M. (2021). La enseñanza híbrida mediante flipped classroom en la educación superior. Revista de Educación Superior.

[11] UNESCO El Coronavirus COVID-19 y la Educación Superior: IMPACTO y Recomendaciones. https://www.iesalc.unesco.org/2020/04/02/el-coronavirus-covid-19-y-la-educacion-superior-impacto-y-recomendaciones/.

[12] Graham C.R., Bonk C.J., Graham C.R. (2006). Blended learning systems: Definitions, current trends and future directions. The Handbook of Blended Learning: Global Perspectives, Local Designs.

[13] Area-Moreira M., Bethencourt-Aguilar A., Martín-Gómez S., Nicolás-Santos M.B. (2021). Análisis de las políticas de enseñanza universitaria en España en tiempos de COVID-19. La presencialidad adaptada. RED.

[14] Osorio L.A., Duart J.M. (2011). Análisis de la interacción en ambientes híbridos de aprendizaje. Comunicar.

[15] Torres M.C.C., Pérez D.A.P., Murillo A.J.A., Plazas N.J.C., Riveros R.A.M. (2021). Modelo instruccional Blended-Flipped: Personalización, flexibilización y metacognición para la nivelación en inglés en la educación superior. Folios.

[16] Hodges C.B., Moore S., Lockee B.B., Trust T., Bond M.A. (2020). The Difference Between Emergency Remote Teaching and Online Learning. Educ. Rev..

[17] García-Planas M.I., Torres J.T. (2020). The transition from the classroom to non-classroom teaching at the UPC during the COVID-19 pandemic. Int. J. Educ. Res. Innov..

[18] Shafaq S., Ali A., Memona F., Ahman A., Soomro A. (2021). Online learning during the COVID-19 pandemic: Applying the self-determination theory in the ‘new normal’. Revista de Psicodidáctica.

[19] Cueva M.A.L., Terrones S.A.C. (2020). Repercusiones de las clases virtuales en los estudiantes universitarios en el contexto de la cuarentena por COVID-19: El caso de la PUCP. Propósitos y Representaciones.

[20] Fardoun H., Yousef M., González-González C., Collazos C.A. (2020). Estudio exploratorio en Iberoamérica sobre procesos de enseñanza-aprendizaje y propuesta de evaluación en tiempos de pandemia. Educ. Knowl. Soc..

[21] Gil-Villa F., Urchaga J.D., Sánchez-Fdez A. (2020). Proceso de digitalización y adaptación a la enseñanza no presencial motivada por la pandemia de COVID-19: Análisis de la percepción y repercusiones en la comunidad universitaria. Revista Latina de Comunicación Social.

[22] Chakraborty P., Mittal P., Gupta M.S., Yadav S., Arora A. (2021). Opinion of students on online education during the COVID-19 pandemic. Hum. Behav. Emerg. Technol..

[23] Aguilera-Hermida P. (2020). College students’ use and acceptance of emergency online learning due to COVID-19. International. J. Educ. Res. Open.

[24] Lembani R., Gunter A., Breines M., Dalu M.T.B. (2020). The same course, different access: The digital divide between urban and rural distance education students in South Africa. J. Geogr. High. Educ..

[25] Parpala A., Katajavuori N., Haarala-Muhonen A., Asikainen H. (2021). How Did Students with Different Learning Profiles Experience ‘Normal’ and Online Teaching Situation during COVID-19 Spring?. Soc. Sci..

[26] Rodríguez-Nogueira O., Leiros-Rodríguez R., Quiroga-Sánchez E., Álvarez-Álvarez M., Álvarez-Barrio L. (2021). Perceptions and Degree of Satisfaction with the Health Sciences University Educational Community Regarding the Measures Adopted for the Prevention of COVID-19 in the Academic Year 2020/2021. Eur. J. Investig. Health Psychol. Educ..

[27] Mishra L., Gupta T., Shree A. (2020). Online teaching-learning in higher education during lockdown period of COVID-19 pandemic. Int. J. Educ. Res. Open.

[28] Diaz M., Walsh B. (2020). Telesimulation-based education during COVID-19. Clin. Teach..

[29] Vasiliadou R. (2020). Virtual laboratories during coronavirus (COVID-19) pandemic. Biochem. Mol. Biol. Educ..

[30] Baticulon R.E., Sy J.J., Alberto N.R.I., Baron M.B.C., Mabulay R.E.C., Rizada L.G.T., Tiu C.J.S., Clarion C.A., Reyes J.C.B. (2021). Barriers to Online Learning in the Time of COVID-19: A National Survey of Medical Students in the Philippines. Med. Sci. Educ..

[31] Bdair I.A. (2021). Nursing students’ and faculty members’ perspectives about online learning during COVID-19 pandemic: A qualitative study. Teach. Learn. Nurs..

[32] Gómez G.M., de los Miró M.Á., Stratta A.E., Mendoza A.B.M.A.B., Zingaretti L. (2020). La Educación superior en tiempos del COVID-19: Análisis comparativo México-Argentina. Revista de Investigación en Gestión Industrial Ambiental Seguridad y Salud en el Trabajo–GISST.

[33] Savage M.J., James R., Magistro D., Donaldson J., Healy L.C., Nevill M., Hennis P.J. (2020). Mental health and movement behaviour during the COVID-19 pandemic in UK university students: Prospective cohort study. Ment. Health Phys. Act..

[34] Ruichen J. (2020). Knowledge, attitudes, and mental health of university students during the COVID-19 pandemic in China. Child. Youth Serv. Rev..

[35] Essadek A., Rabeyron T. (2020). Mental health of French students during the Covid-19 pandemic. J. Affect. Disord..

[36] Saravia-Bartra M.M., Cazorla-Saravia P., Cedillo-Ramirez L. (2020). Nivel de ansiedad de estudiantes de medicina de primer año de una universidad privada del Perú en tiempos de Covid-19. Rev. Fac. Med. Hum..

[37] Wang C., Zhao H. (2020). The Impact of COVID-19 on Anxiety in Chinese University Students. Front. Psychol..

[38] Charbonnier E., Le Vigouroux S., Goncalves A. (2021). Psychological Vulnerability of French University Students during the COVID-19 Pandemic: A Four-Wave Longitudinal Survey. Int. J. Environ. Res. Public Health.

[39] Kaparounaki C.K., Patsali M.E., Mousa D.P.V., Papadopoulou E.V., Papadopoulou K.K., Fountoulakis K.N. (2020). University students’ mental health amidst the COVID-19 quarantine in Greece. Psychiatry Res..

[40] Sarmiento Á.S., Ponce R.S., Bertolín A.G. (2021). Resilience and COVID-19. An Analysis in University Students during Confinement. Educ. Sci..

[41] Hasan N., Bao Y. (2020). Impact of “e-learning crack-up” perception on psychological distress among college students during COVID-19 pandemic: A mediating role of “fear of academic year loss”. Child. Youth Serv. Rev..

[42] Biswas B., Roy S.K., Roy F. (2020). Students Perception of Mobile Learning during COVID-19 in Bangladesh: University Student Perspective. Aquademia.

[43] Bryman A. (2006). Integrating quantitative and qualitative research: How is it done?. Qual. Res..

[44] León O., Montero I. (2002). Métodos de Investigación en Psicología y Educación.

[45] Bisquerra R. (2014). Metodología de la Investigación Educativa.

[46] Barroso J., Cabero J. (2010). La Investigación Educativa en TIC. Visiones Prácticas.

[47] Cabero J., Llorente M.C. (2013). La aplicación del juicio de experto como técnica de evaluación de las tecnologías de la información y comunicación (TIC). Revista de Tecnología y Comunicación en Educación.

[48] Aiken L. (1985). Three Coeficients for Analyzing the Reliability and Validity of Ralings. Educ. Psychol. Meas..

[49] Merino C., Livia J. (2009). Intervalos de confianza asimétricos para el índice la validez de contenido: Un programa Visual Basic para la V de Aiken. Anales de Psicología.

[50] Penfield R.D., Giacobbi P.R. (2004). Applying a score confidence interval to Aiken’s item content-relevance index. Meas. Phys. Educ. Exerc. Sci..

[51] George D., Mallery P. (2003). SPSS for Windows Step by Step: A Simple Guide and Reference. 11.0 Update.

[52] Nassr R.M., Aborujilah A., Aldossary D.A., Aldossary A.A.A. (2020). Understanding education difficulty during COVID-19 lockdown: Reports on malaysian university Students’ experience. IEEE Access.

[53] Rosario-Rodríguez A., González-Rivera J.A., Cruz-Santos A., Rodríguez-Ríos L. (2020). Demandas Tecnológicas, Académicas y Psicológicas en Estudiantes Universitarios durante la Pandemia por COVID-19. Revista Caribeña de Psicolgía.

[54] Cao W., Fang Z., Hou G., Han M., Xu X., Dong J., Zheng J. (2020). The psychological impact of the COVID-19 epidemic on college students in China. Psychiatry Res..

[55] García-Peñalvo F.J. (2020). Modelo de referencia para la enseñanza no presencial en universidades presenciales. Campus Virtuales.

[56] Shah S.S., Shah A.A., Memon F., Kemal A.A., Soomro A. (2021). Aprendizaje en línea durante la pandemia de COVID-19: Aplicación de la teoría de la autodeterminación en la “nueva normalidad”. Revista de Psicodidáctica.

[57] Fernández P., Vergara D. (2020). Aprendizaje virtual en tiempos de COVID-19: Opinión del alumnado universitario. Eduweb.

[58] Bosch M.A.S.J., Núñez R.D.G., Villar N.M., Hernández A.F., Brito A.D. (2020). Experiencias y alternativas académicas de la Universidad de Ciencias Médicas de Cienfuegos durante la COVID-19. Medisur.

[59] Ragusa A.T. (2017). Technologically mediated communication: Student expectations and experiences in a FOMO society. Int. J. Educ. Technol. High. Educ..

[60] Duncan K., Kenworthy A., McNamara R. (2012). The effect of synchronous and asynchronous participation on students’ performance in online accounting courses. Account. Educ. Int. J..

[61] Pavla S., Hana V., Jan V. (2015). Blended Learning: Promising strategic alternative in higher education. Procedia-Social and Behavioral Sciences. Procedia Soc. Behav. Sci..

[62] Sriarunrasmee J., Techataweewan W., Mebusaya R. (2015). Blended Learning Supporting Self-Directed Learning and Communication Skills of Srinakharinwirot University’s First Year Students. Procedia Soc. Behav. Sci..

[63] Klimova B., Kacetl J. (2015). Hybrid learning and its current role in the teaching or foreign languages. Procedia Soc. Behav. Sci..

[64] De Anda A.B.B., González C.Á., Herrera A.M.B., Cadena M.J.C., Valenzuela R.G. (2021). Ambientes híbridos de aprendizaje en estudios de posgrado. Revista Iberoamericana de Tecnología en Educación y Educación en Tecnología.

[65] Pérez-López E., Vázquez A., Cambero S. (2020). Educación a distancia en tiempos de COVID-19: Análisis desde la perspectiva de los estudiantes universitarios. RIED Revista Iberoamericana de la Educación Digital.

[66] Olmos-Gómez M.D.C., Luque-Suárez M., Ferrara C., Cuevas-Rincón J.M. (2021). Quality in Higher Education and Satisfaction among Professors and Students. Eur. J. Investig. Health Psychol. Educ..

